# Supplementary material for: Single Nucleotide Polymorphism Discovery and Genetic Differentiation Analysis of Geese Bred in Poland, Using Genotyping-by-Sequencing (GBS)
Source: Genes (Basel). 2021 Jul 14;12(7):1074. doi: 10.3390/genes12071074 (PMC8307914; doi:10.3390/genes12071074)
Supplement: Supplementary file 1 [file genes-12-01074-s001.zip › Supplementary File 4.html]

JASP 


# Results

## Descriptive Statistics

| Descriptive Statistics | | | | | | | | | | | | | | | | | | | | | | | | | | | |
| --- | --- | --- | --- | --- | --- | --- | --- | --- | --- | --- | --- | --- | --- | --- | --- | --- | --- | --- | --- | --- | --- | --- | --- | --- | --- | --- | --- |
|  | | Ga | | Po | | Ry | | Ki | | La | | BK | | Pd | | Sl | | Ro | | Lu | | Ku | | Su | | Ka | |
| Valid |  | 698 |  | 760 |  | 730 |  | 697 |  | 755 |  | 791 |  | 760 |  | 783 |  | 753 |  | 765 |  | 581 |  | 733 |  | 644 |  |
| Missing |  | 93 |  | 31 |  | 61 |  | 94 |  | 36 |  | 0 |  | 31 |  | 8 |  | 38 |  | 26 |  | 210 |  | 58 |  | 147 |  |
| Mean |  | 0.816 |  | 0.812 |  | 0.816 |  | 0.823 |  | 0.814 |  | 0.815 |  | 0.810 |  | 0.816 |  | 0.810 |  | 0.812 |  | 0.823 |  | 0.813 |  | 0.819 |  |
| Median |  | 0.910 |  | 0.885 |  | 0.890 |  | 0.890 |  | 0.890 |  | 0.880 |  | 0.880 |  | 0.890 |  | 0.880 |  | 0.890 |  | 0.930 |  | 0.890 |  | 0.890 |  |
| Std. Deviation |  | 0.237 |  | 0.206 |  | 0.209 |  | 0.197 |  | 0.211 |  | 0.188 |  | 0.201 |  | 0.212 |  | 0.205 |  | 0.209 |  | 0.249 |  | 0.212 |  | 0.219 |  |
| Variance |  | 0.056 |  | 0.042 |  | 0.044 |  | 0.039 |  | 0.045 |  | 0.035 |  | 0.040 |  | 0.045 |  | 0.042 |  | 0.044 |  | 0.062 |  | 0.045 |  | 0.048 |  |
| Minimum |  | 0.000 |  | 0.050 |  | 0.000 |  | 0.000 |  | 0.020 |  | 0.040 |  | 0.020 |  | 0.000 |  | 0.000 |  | 0.030 |  | 0.000 |  | 0.050 |  | 0.000 |  |
| Maximum |  | 1.000 |  | 1.000 |  | 1.000 |  | 1.000 |  | 1.000 |  | 1.000 |  | 1.000 |  | 1.000 |  | 1.000 |  | 1.000 |  | 1.000 |  | 1.000 |  | 1.000 |  |
|  | | | | | | | | | | | | | | | | | | | | | | | | | | | |

### Distribution Plots

#### Ga

#### Po

#### Ry

#### Ki

#### La

#### BK

#### Pd

#### Sl

#### Ro

#### Lu

#### Ku

#### Su

#### Ka
